# Compulsory medical intervention versus external constraint in pandemic control

**DOI:** 10.1136/medethics-2020-106435

**Published:** 2020-08-20

**Authors:** Thomas Douglas, Lisa Forsberg, Jonathan Pugh

**Affiliations:** 1 Oxford Uehiro Centre for Practical Ethics, Oxford University, Oxford, Oxfordshire, UK; 2 Jesus College, Oxford, Oxfordshire, UK; 3 Faculty of Philosophy, University of Oxford, Oxford, Oxfordshire, UK; 4 Faculty of Law, Oxford University, Oxford, Oxfordshire, UK; 5 Somerville College, Oxford, Oxfordshire, UK

**Keywords:** public health ethics, law, ethics, vaccination, compulsion, mental health law, quarantine, public health law, isolation

## Abstract

Would compulsory treatment or vaccination for COVID-19 be justified? In England, there would be significant legal barriers to it. However, we offer a conditional ethical argument in favour of allowing compulsory treatment and vaccination, drawing on an ethical comparison with external constraints—such as quarantine, isolation and ‘lockdown’—that have already been authorised to control the pandemic in this jurisdiction. We argue that, *if* the permissive English approach to external constraints for COVID-19 has been justified, then there is a case for a similarly permissive approach to compulsory medical interventions.

Governments worldwide have responded to the COVID-19 pandemic with sweeping constraints on freedom of movement and association, ranging from isolation of confirmed cases, to quarantine of individuals thought to have been exposed to the virus, to general lockdowns requiring all individuals to stay at home except for specified purposes.

To guarantee the lawfulness of these measures, and others that might become necessary, governments have introduced a range of new legal instruments. But in many countries, one measure that these instruments leave off the table is the use of compulsory medical interventions, by which we mean physically invasive treatments or vaccinations. In the near-term future, the most pressing moral issue raised by newly developed vaccines for COVID-19 will likely concern fair distribution, since these vaccines will initially be a scarce resource.^1^ However, once such vaccines or treatments for COVID-19 become widely available, another issue will come to the fore. There may be considerable political interest in enforcing the uptake of treatments and vaccines, since this could allow for the quickest and safest route out of remaining ‘lockdown’ and distancing arrangements. In the case of vaccines, there will be a need to ensure that enough people are vaccinated to confer herd immunity. There may also be an argument for ensuring that people who have contact with many others, such as teachers, retail staff and healthcare workers, are vaccinated without exception. In the case of treatments, we might hope that widespread use of antiviral therapies could reduce the burden on health services by reducing the number of infected individuals who require intensive care.

This raises the question: would compulsory medical intervention for COVID-19 be justified?^1^ In the first part of this article, we show that, in England,^2^ there would be significant legal barriers to it. But in the second part, we present a conditional ethical case for seeking to overcome those barriers. We argue that, *if* the permissive English approach to external constraints for COVID-19 has been justified, there is at least a defeasible case for permitting some compulsory medical interventions. This is because, legal barriers aside, it is morally no harder to justify safe, effective and only moderately invasive compulsory medical interventions for COVID-19 than some of the external constraints that have already been authorised for the control of the condition in England.

## Barriers to compulsory medical intervention for COVID-19 in English law

In English law, the competent individual’s right to refuse any medical intervention that interferes with her body is well-established and enjoys strong protection. As we shall see, mental health law provides some exceptions to this right,^3^ but for most individuals who possess decision-making capacity, the right persists even when the individual’s reasons for refusing an intervention are bizarre, irrational or non-existent, when undergoing the intervention would clearly be in her best interests, and indeed when refusing the intervention would certainly lead to her death.^2–5^

Case law of the European Court of Human Rights suggests that the individual’s right to make her own medical decisions, and in particular to refuse interventions that interfere with her body, is within the ambit of the right to private life protected by article 8 of the European Convention on Human Rights (ECHR).^6–8^ The UK is party to the ECHR and has incorporated it into English law via the Human Rights Act 1998. Any non-consensual medical intervention that interferes with recipients’ bodies would likely engage article 8 ECHR. In *X v Austria,* the European Court of Human Rights (ECtHR) held that ‘[c]ompulsory medical intervention, even if it is of minor importance, must be considered an interference with [article 8 ECHR]’.^9^

The protection that article 8 ECHR offers for individuals’ bodily integrity is not absolute, however. Interferences can be justified if they are in accordance with national law, pursue a legitimate aim and are proportionate in relation to this aim. In the case of vaccinations or treatments intended to stem the spread of a pandemic disease, a legitimate aim is present; the ECtHR has previously accepted non-consensual blood tests, vaccinations and screening programmes as justified on grounds of, *inter alia*, protection of the rights and freedoms of others,^9^ and public safety.^10^ But non-consensual interventions that interfere with individuals’ bodily integrity may fail the proportionality test. One reason that they may do so is that other equally effective and less restrictive alternative measures are available. Yet even if no other equally effective alternative measures exist, it might be argued that non-consensual medical interventions for COVID-19 would be disproportionate, for example, because the benefits are insufficiently important to justify the infringement of bodily integrity that they would entail.^4^ We shall challenge this view in the second half of this paper.

Prior to the pandemic, English public health law, like medical and human rights law, was not hospitable to compulsory medical intervention for pandemic control. Section 45G(2) of the Public Health (Control of Disease) Act 1984 (as amended by the Health and Social Care Act 2008) authorises a Justice of the Peace to order one or more of 11 of restrictions of liberty on a potentially infectious person, when that is necessary to remove or reduce the risk of their transmitting a pathogen that poses a significant harm to human health.^11^ However, although the listed restrictions include the imposition of medical examination (s 45G(2)(a)) and monitoring (s 45G(2)(h)), they do not include physically invasive therapeutic or preventive interventions.

Moreover, although Section 45C of the Act grants the Secretary of State the power to pass further domestic regulations deemed necessary to prevent the spread of an infectious disease,^5^ section 45E prohibits these regulations from including provisions to directly impose compulsory medical treatment (including vaccination) at a population level.^6^

The powers that may be exercised in the interests of public health have, however, changed with the Coronavirus Act 2020.^12^ This act extends the power to impose public health restrictions beyond Justices of the Peace to the relevant Secretary of State, and designated public health officers. Moreover, Schedule 21 of the Act explicitly authorises the imposition of invasive testing methods, including the withdrawal of blood samples and respiratory secretions for the purposes of screening and assessment. As with the earlier legislation, compulsory treatment is not included in the Coronavirus Act among the requirements that may be imposed in order to prevent the spread of infections. However, the Act arguably opens the door to such treatment. Whereas the Public Health Act states that a Justice of the Peace may impose ‘one or more of the following’ 11 restrictions listed in section 45G(2) (the list thus appearing to be exhaustive), Schedule 21, paragraph 14(1 - 2) of the Coronavirus Act simply states that if screening confirms that an individual is infected with coronavirus, public health officers may impose ‘such requirements and restrictions on the person as the officer considers necessary and proportionate’. This wording arguably leaves the door to compulsory medical interventions for COVID-19 somewhat ajar.^7^

However, before such interventions could be considered, the significant barriers posed by medical and human rights law would need to be overcome. In what follows, we present an ethical case for seeking to overcome those barriers.

## An ethical argument for permitting compulsory medical intervention for COVID-19

Ethicists have argued in favour of compulsory vaccination on diverse theoretical grounds, including by appealing to libertarian principles governing the imposition of unjust harm or risk of harm on others,^13^ to collective self-defence^14^ and to moral duties of easy rescue or fairness.^15–17^ However, such arguments will only receive as much support as the sometimes controversial moral theories underlying them. Thus, rather than invoking any of these particular principles or duties here, we instead offer an argument that we hope can be more broadly accepted. The argument has a conditional and comparative form. We will argue that compulsory medical interventions for the control of COVID-19 would be ethically preferable, or at least not dispreferable, to some forms of external constraint—such as quarantine, isolation or ‘lockdown’—that English law already authorises for the same purpose. This implies that, *if* allowing these forms of external constraint has been justified, then there is a case (though not necessarily a decisive one) for allowing compulsory medical intervention as well.^8^

We begin with a point about harm: at least for reasonably safe medical interventions—interventions that can be expected to pose no greater risk of harm to the individual than typical, widely used treatments and vaccines—compulsory medical intervention is likely to impose no more (and perhaps substantially less) harm on those subjected to it than do the types of external constraints being deployed currently. Many of us would prefer being required to undergo a safe treatment or vaccination to being subjected to constraints on movement for an extended period so, if well-being is determined by preference satisfaction, compulsory medical intervention would, for many of us, be less harmful than external constraint.

Hedonistic understandings of well-being yield a similar result; mandating a safe treatment or vaccine could, for many people, be expected to cause less experiential suffering than severe constraints on movement. A recent evidence review suggests that quarantine is associated with significant negative psychological effects including post-traumatic stress symptoms, confusion and anger, and there is some evidence to suggest that some of these effects can last for years after quarantine.^18^ There have also been reports of increased rates of domestic abuse under lockdown arrangements.^19^ By contrast, vaccinations typically have few side effects, and severe side effects are usually vanishingly rare.^20^ It is, of course, quite likely that the first-developed treatments and vaccines for COVID-19 will be less safe than established vaccines, given the rapid development process. However, their safety might still be comparable to many widely used pharmaceutical interventions, which are often somewhat less safe than vaccines. Moreover, it is quite likely that early *treatments* for COVID-19 will be interventions that are already employed for other conditions, and whose general safety is well established. It might be argued that, to the side effects of medical interventions for COVID-19, we need to add the side effects of making these interventions compulsory. Perhaps the compulsion involved in compulsory vaccination or treatment would cause significant distress. However, concerns about such distress could be avoided by exempting individuals with particularly strong objections from the requirement to undergo treatment or vaccination; we are not defending the view that compulsion should be universal.

What, then, is the argument for thinking that compulsory medical interventions are more morally problematic than external constraints? The most promising argument, we think, appeals to the putative right to bodily integrity, understood as a right against (certain forms of) significant bodily interference. This right is normally understood to protect even against safe and beneficial forms of bodily interference, and this right is, many would claim, stronger—in the sense that it is typically harder to justify its infringement—than our rights to freedom of movement and association—the rights imperilled by external constraint.

But the view that the right to bodily integrity lies on a plane above rights to free movement and association can be challenged.^21^ Dominant philosophical defences of the right to bodily integrity often appeal to the concept of the self or the person. For example, some see the right to bodily integrity as an implication of the right to self-ownership: we own our selves, our selves include our bodies, therefore we own our bodies, and our property rights in our bodies include rights against interference by others.^22^ Others see the right as an implication of our personal sovereignty—understood on analogy with the sovereignty of a state over its territory.^23^ One way to defend the special strength of the right to bodily integrity, then, would be to appeal to the close relationship between the body and the self or person. Some would say that we *are* our bodies—or at least, we share our physical boundaries with them.^24 25^ Others would deny this but nevertheless maintain that there is some special and close relationship between us—ourselves or our persons—and our bodies.^26^

Note, however, that there is also a special and close relationship between the self and one’s loved ones and the self and one’s immediate physical environment. Proponents of the extended mind thesis hold that our mind resides as much in external cognitive aids, such as our diaries, books and smartphones as in our brains.^27–29^ This arguably implies that these external objects are *part of* our selves.^9^ But even those who deny that such objects are literally part of ourselves are likely to concede that our selves rely heavily on them. They are also likely to concede that our selves are highly dependent—for both their persistence and flourishing—on our closest social relationships. Indeed, there has been a prominent ‘relational turn’ in our understanding of both the self, and the concept of self-governance or autonomy, in recent scholarship.^30 31^

Suppose that our closest relationships and surroundings are just as important to the self as our bodies. Suppose, moreover, that the importance of the body to the self is indeed what justifies the peculiar strength of the right to bodily integrity. Since our closest relationships and surroundings are severely affected by restrictions of freedom of movement and association, it may follow that our rights to freedom of movement and association are just as strong as the right to bodily integrity.

However, even if this is incorrect—even if the right to bodily integrity is indeed stronger than rights to freedom of movement and association, in the sense that infringements of bodily integrity are normally harder to justify than restrictions on movement and association—it will not straightforwardly follow that medical interventions for the purpose of pandemic control are *always* harder to justify than external constraint for the same purpose. It is important to attend also to the nature and severity of the rights infringement. Both rights to bodily integrity and rights to free movement and association are plausibly *graded* rights, in the sense that they provide stronger protection against more severe interferences, and weaker protection against less severe interferences. Most of us would think, for example, that though non-sexual touching can infringe the right to bodily integrity, it typically involves a less serious infringement of the right, and is thus easier to justify, than a more severe interference, such as cutting a person with a knife. Similarly, most of us would think that a mild interference with freedom of movement and association, such as a probation order requiring a person to present to a probation office every month, is easier to justify than a severe interference, such as solitary confinement.

This is relevant because at least some of the external constraints currently being lawfully employed to control the COVID-19 pandemic—such as isolation in a hospital facility—surely involve a *very severe* interference with freedom of movement and association. On the other hand, requiring a person to receive a single vaccination by injection, say, would arguably involve only a moderate interference with bodily integrity. Thus, even if there are reasons to think that infringing bodily integrity is—other things being equal—harder to justify than infringing freedom of movement and association, other things may not be equal. A difference in the typical strength of these rights might be outweighed by a difference in the severity of the interference that infringes them.^10^

There is also a third and final difficulty faced by the appeal to bodily integrity. Someone might claim that, *contra* our first argument, the right to bodily integrity *is* stronger than rights to freedom of movement and association. Moreover, they might claim that the difference in strength is so great that even a moderate interference with bodily integrity is harder to justify than a severe interference with freedom of movement and association. However, this view would be difficult to square with English law on two types of bodily interference that are somewhat analogous to, though distinct from, compulsory treatment or vaccination for pandemic control.

The first is law on medical *testing* for the purposes of pandemic control. As we explained above, the Coronavirus Act 2020 already allows compulsory testing for COVID-19, which is significantly invasive (it involves insertion of a swab into the throat). It is not clear that compulsory injection of a vaccine, say, would involve a substantially more severe bodily interference than such testing. True, the vaccination would introduce a biologically active agent that is then disseminated throughout body. But testing may need to be repeated, in the case of non-infected individuals many times, whereas vaccination would most likely be a one-off injection. Moreover, future testing could involve the withdrawal of blood samples and respiratory secretions.^12^ The removal of such biological substances from the body might reasonably be thought as severe, as an instance of bodily interference, as introducing a tiny amount of vaccine. It might, of course, be argued that compulsory vaccination risks greater harm than mandatory testing; complications of vaccination, even if extremely rare, can lead to death. However, if risk of harm is the issue, then some already authorised forms of external constraint will be yet harder to justify since the risks of harms they pose are even greater: recall the abovementioned effects of lockdown on mental health and domestic abuse.^32^

A second area of English law that allows for significant interference with bodily integrity is mental health law. In England, the Mental Health Act 1983 (as amended by the Mental Health Act 2007) allows both detention *and certain forms of non-consensual treatment* of individuals with mental disorders when this is deemed necessary for the health or safety of the patient or for the protection of other persons, even if the individual has decision-making capacity.^33^ Non-consensual treatment of capacitous individuals under the Mental Health Act for the purposes of the protection of others is analogous to the case of compulsory medical intervention for pandemic control. The Mental Health Act does include significant restrictions on the use of compulsory treatment. For example, the threat that the individual poses to others (or themselves) in the absence of treatment must be imminent, and the treatment must be appropriate to the patient’s condition.^11^ However, we see no reason why similar conditions could not be placed on—and quite commonly satisfied with respect to—compulsory medical intervention for pandemic control. It is thus difficult to see how the current approach to mental health—which places compulsory medical intervention and external constraint roughly on a par—could be reconciled with the strong preference given to external constraint in the case of pandemic control.

Indeed, if anything, it might seem that considerations of harm suggest that we should be *more* willing to interfere with people’s bodies in the case of pandemic control than in mental health. The risk of extensive harm associated with not employing medical intervention in the case of COVID-19, for instance, is likely to be greater than in typical mental health cases. Consider that, prior to the lockdown, individuals with COVID-19 were, on average, infecting at least two other people during their infectious period, with each of those individuals infecting the same number again, and so on.^34^ With around 7 days between becoming infected and one’s peak infectiousness to others, this means that a single infected individual might, over the course of, say, 10 weeks have led to the infection of over 1000 people,^12^ of whom somewhere between 2 and 20 would be expected to die of the disease.^13^ We do not know how these figures will change as lockdown and distancing rules are fully relaxed; presumably infection rates will be controlled to some extent by basic hygiene. Still, this back-of-the-envelope calculation suggests that vaccinating a single individual with a particularly large number of contacts (a retail worker, say) might be expected to prevent several deaths over a relatively short period. It would also, of course, prevent significant morbidity since, in addition to the risk of death, COVID-19 carries risks of, for example, (potentially permanent) organ damage, the magnitude of which is yet unknown.^35 36^ Some individuals detained under mental health law might pose serious threats to others’ health, but risks of a similar magnitude to COVID-19 would be very rare.

On the other hand, the harm associated with compulsory medical intervention in the case of vaccination for COVID-19 is likely to be less than in many mental health cases. Vaccination would most likely be a one-off intervention, and it is of a kind that many people routinely undergo voluntarily without giving the matter much thought. The same could not be said of some mental health treatments—such as lithium for bipolar disorder and antipsychotics for schizophrenia—that are quite commonly imposed under mental health legislation.

## Concluding thoughts

We have argued that compulsory medical intervention for the control COVID-19 would not be harder to justify, morally, than some forms of external constraint that are already being used, or have been authorised, for this same purpose.

Our arguments invoked two chief values: harm and bodily integrity. With respect to harm, compulsory medical interventions will typically be less harmful to those subjected to them than some of the external constraints currently being implemented in response to the COVID-19 pandemic. With respect to bodily integrity: in the first place, it is doubtful that the right to bodily integrity is any stronger than the rights to free movement and association engaged by external constraint; in the second place, free movement and association are severely constrained by measures such as quarantine and isolation, whereas compulsory vaccination or treatment would likely involve only a moderate interference with bodily integrity; and in the third place, the strong precedence given to bodily integrity in the case of treatments and vaccines for pandemic control is difficult to reconcile with existing law on *testing* for pandemic disease and on mental health treatments.

What, practically speaking follows from our argument? One possibility is that nothing follows, since the law need not always reflect morality; there can be perfectly good pragmatic or political reasons for regulating one type of intervention more stringently than another, even though the interventions are similar in their moral justifiability. We take it, however, that there is at least a defeasible case in favour of laws that treat morally similar practices similarly. Thus, our argument implies that there is at least a case for bringing law on external constraint for pandemic control and law on compulsory medical intervention for the same purpose into line. One way to do this, of course, would be to regulate the use of external constraints more stringently. Perhaps the law currently permits quarantine, isolation and lockdown too easily. But for those of us who find that hard to accept, the other possibility may be more attractive: perhaps current legal constraints on compulsory medical intervention ought to be loosened.^14^

To be clear, if these constraints were indeed to be loosened, safeguards would need to be put in place to ensure that medical interventions are imposed only when safe, effective and necessary, and where the degree of physical invasion that they involve is not too great. In some cases, compulsory medical intervention might be unnecessary simply because there are means short of compulsion for ensuring that (a sufficient number of) people undergo the intervention. Vaccine certification might, for example, be sufficient.^37^ Moreover, if compulsion were to be introduced, exceptions would need to be built in for those who are likely to suffer side effects, and—perhaps—for those who have strong moral objections or simply prefer to lower their risk to others through other means. Although we cannot defend it in full here, we think that one promising option would be for the government to offer the choice: ‘either have yourself vaccinated, or stay at home’. That would treat external constraint and medical intervention as on a par, while giving individuals greater freedom than in a situation where either external constraints or medical interventions are imposed.

## Data Availability

There are no data in this work.

## Footnotes

For discussion of what it means for a vaccination policy to be ‘compulsory’, see^38–41^.

Health is in the United Kingdom a devolved matter, and England and Wales therefore have separate public health law— including infectious disease control—regimes. We therefore focus just on England here, even though many of our arguments also apply to the public health regimes in other jurisdictions in the United Kingdom and elsewhere.

A patient’s consent will be valid when (i) she possesses decision-making capacity;^42^ (ii) she possesses sufficient information about the intervention;^43^ and (iii) her decision is not unduly influenced by external influences.^2^ The only exception to this is individuals detained under mental health law, who may, when certain conditions are met, receive interventions without their consent even when they possess decision-making capacity.

Whether a given measure is ‘necessary in a democratic society is determined by balancing the interests of the individual subjected to the measure against those of the Member State subjecting her to it. In order for an interference with individual rights to be ‘necessary’, there has to be a pressing social need for it. Member States are afforded a margin of appreciation in determining whether such a need exists, subject to review by the ECtHR. The Court will take into account factors such as the relevance and sufficiency of the reasons given by the Member State by way of justification.^44 45^ Proportionality is determined taking into account the legislative choices made and decision-making process followed by the Member State, the parliamentary and judicial reviews undertaken, and procedural safeguards available to individuals subjected to the measure. The Member State’s decision-making process in employing the measure must be fair and give due attention to the safeguarding of the article 8 interests of individuals subjected to it.^46^ In the case of compulsory medical intervention for Covid-19, Member State’s interests in safeguarding the lives and health of others seem compelling, so give that sufficient safeguarding mechanisms were put in place, it does not seem unreasonable to assume that such interventions could be justified. It is also not obvious that the status quo approach would be accepted as proportionate. For an argument to the effect that the (pre-Covid-19) UK public health law framework ‘fails to set out a clear and proportionate approach’ in respect of vaccination, see^38^.

This power was used recently to create the Coronavirus Regulations,^47^ which preceded the Coronavirus Act^12^.

Notably though, this provision may not preclude the possibility that such regulations could authorise compulsory treatment on a case-by-case basis.^48^

Paragraph 14(3) of the Coronavirus Act 2020^12^ provides some examples of interventions that this may include, but there is no reason to think that this list is exhaustive. However, although compulsory treatment is neither expressly prohibited or permitted under the act, we believe that there are significant difficulties with interpreting the law as implicitly permitting compulsory treatment. Both the presumption of liberty in public law, and the fact that the Public Health Act prohibits compulsory treatment in future regulations authorised under its auspices, raise considerable obstacles for this interpretation.

For similar arguments made in the context of criminal justice, see^21^. For the suggestion that such an argument might be made with respect to Covid-19, see^49 50^.

cf.^51^

Also relevant here is the degree of compulsion or coercion involved. An interference with the body could be easier to justify than a constraint on free movement and association because, for example, there are more exception clauses allowing individuals to escape the former than the latter. We might imagine, for example, that individuals could be exempted from vaccination on religious grounds, though they could not be exempted from quarantine on the same grounds. We thank an anonymous reviewer for pressing us to consider this point. For a discussion of different public health mechanisms with different degrees of compulsion, see^38^.

Moreover, if three months or more have elapsed since the first occasion (in the period for which the patient is liable to be detained) when certain medical treatments have been administered to the patient (by any means for their mental disorder), the lawful non-consensual imposition of non-urgent treatment after that point will require approval from a second opinion appointed doctor (s 58(b).

Each infected individual infects 2 others and that there are 10 cycles of infection within a 10 week period, one initially infected individual would lead to the infection of 210 = 1,024 people by the end of the period.

Current estimates for the mortality rate of Covid-19 typically place it between 0.2% and 2%.

Interestingly, Germany appears to be moving in this direction in the case of vaccinations for measles. In March 2020, Germany made vaccinations against measles compulsory as a condition for children’s day care and school attendance.^52^
